# The clinical significance of sarcopenia in patients with hepatocellular carcinoma treated with lenvatinib and PD-1 inhibitors

**DOI:** 10.3389/fimmu.2024.1380477

**Published:** 2024-04-18

**Authors:** Wei Sun, Xue Yin, Xiaomin Liu, Jianying Wei, Minghua Yu, Wendong Li, Xiaoyan Ding, Jinglong Chen

**Affiliations:** Department of Cancer Center, Beijing Ditan Hospital, Capital Medical University, Beijing, China

**Keywords:** sarcopenia, HCC, PD-1 inhibitor, lenvatinib, psoas muscle index

## Abstract

**Background and aim:**

Sarcopenia has gained considerable attention in the context of hepatocellular carcinoma, as it has been correlated with a poorer prognosis among patients undergoing sorafenib or lenvatinib treatment for hepatocellular carcinoma (HCC). The clinical significance of sarcopenia in first-line advanced HCC patients treated with lenvatinib and programmed death-1 (PD-1) inhibitors needs to be clarified.

**Methods:**

Sarcopenia was diagnosed using CT (Computed tomography) or MRI (Magnetic Resonance Imaging), with the psoas muscle index (PMI) as the surrogate marker. Patients were grouped based on sarcopenia presences, and a comparative analysis examined characteristics, adverse events, and prognosis. The Cox regression analysis was applied to identify independent prognostic factors for survival, while nomograms were constructed to predict 1-year survival.

**Results:**

Among 180 patients, 46 had sarcopenia. Patients with baseline sarcopenia demonstrated significantly inferior median progression-free survival (mPFS) (3.0 vs. 8.3 months) and median overall survival (mOS) (7.3 vs. 21.6 months). The same results for mPFS (3.3 vs. 9.2 months) and mOS (9.4 vs. 24.2 months) were observed in patients who developed sarcopenia after treatment. Furthermore, significantly higher grade 3 or higher adverse events (AEs) (73.91% vs 41.79%, p<0.001) were recorded in the sarcopenia group compared to the non-sarcopenia group. In the multivariate analysis, distant metastasis, elevated PLR and CRP levels, and low PMI remained independent predictive factors for poor OS. Additionally, skeletal muscle loss remained a significant independent risk factor for PFS. We developed a nomogram incorporating these four indicators, which predicted 12-month survival with a C-index of 0.853 (95% CI, 0.791 – 0.915), aligning well with actual observations.

**Conclusion:**

The prognosis of patients with HCC and sarcopenia is significantly worse when treated with lenvatinib and PD-1 inhibitors. The combination regimen of lenvatinib plus PD-1 inhibitors should be cautiously recommended due to the inferior prognosis and higher AEs.

## Introduction

Hepatocellular carcinoma (HCC) ranks as the sixth most prevalent malignant neoplasm worldwide and stands as the fourth leading cause of cancer-related mortality (1). In China, chronic hepatitis B virus (HBV) infection is a predominant risk factor for HCC, while in Western countries, hepatitis C and lifestyle factors exhibit higher prevalence rates (2). Despite significant advancements in the prevention and diagnosis of HCC, approximately 70% of patients are diagnosed at an advanced stage, necessitating systemic therapy as the standard recommendation. Currently, anti-angiogenic drugs in combination with PD-(L)1 inhibitors have demonstrated remarkable efficacy in advanced HCC (3–6). The Food and Drug Administration (FDA) recommends a combination of Atezolizumab and Bevacizumab (ATEZ/BEV) as the preferred first-line treatment option (3). Moreover, owing to its superior objective response rate (ORR) and significantly prolonged overall survival (OS) in the Asian subgroup, the combination of lenvatinib and PD-(L)1 inhibitors gained widespread utilization in China (7, 8). However, the predictive biomarkers are uncertain, and identifying those who will benefit from this combination regimen remains a critical issue in clinical practice.

Sarcopenia, characterized by diminished muscle strength, skeletal muscle mass, and physical performance, is prevalent among the elderly population and can arise as a consequence of hepatic or renal dysfunction, inflammatory disorders, and malignancies such as HCC (9). In the treatment of HCC, sarcopenia is strongly associated with an unfavorable prognosis. Studies have shown that in systemic treatment of uHCC, sarcopenia is related to adverse clinical outcomes with tyrosine kinase inhibitors (TKIs) such as sorafenib and lenvatinib. The assessment primarily relies on the skeletal muscle index (SMI) (10–17). SMI is recommended as a method of accurate muscle mass assessment, but its complex calculation limits clinical application (18, 19). The psoas muscle index (PMI) offers advantages of being quicker and easier to obtain than SMI, can be an alternative for assessing sarcopenia (20). Fujita et al. (21) found that HCC patients treated with lenvatinib, who experienced a substantial reduction in PMI, exhibited a shorter OS than those with a minor reduction. Moreover, the impact of sarcopenia on HCC patients receiving PD-(L)1 inhibitors remains controversial. While studies have indicated no significant correlation between low skeletal muscle mass (LSMM) and survival rates (22–24), decreased skeletal muscle was significantly associated with poor progression-free survival (PFS) and OS in advanced HCC patients undergoing ATEZ/BEV treatment (25). Despite the widespread recognition and significance of sarcopenia, its precise role and impact under the backdrop of lenvatinib combined with PD-1 inhibitors still need to be completed.

Therefore, the relationship between sarcopenia and clinical outcomes in HCC patients receiving the combination regimen of lenvatinib and PD-1 inhibitors merits further research and exploration. This study aims to explore further and clarify the relationship between sarcopenia and clinical outcomes in HCC patients receiving lenvatinib combined with PD-1 inhibitors.

## Materials and methods

### Study design and patient selection

We retrospectively selected patients with unresectable HCC who received lenvatinib in combination with PD-1 inhibitors at Beijing Ditan Hospital, Capital Medical University, from July 2019 to January 2022. The following patients were included: (1) HCC diagnosed by histological or radiological criteria as defined by the American Association for the Study of Liver Diseases (AASLD) guidelines; (2) age 18 years or older; (3) patients with tolerable general status, Eastern Cooperative Oncology Group score (ECOG PS) 0-2, Child-Pugh class A or B; and (4) received lenvatinib combined with PD-1 inhibitors as the first-line of therapy. The Main exclusion criteria were as follows: (1) patients who received systemic drugs, including sorafenib, lenvatinib, PD-1 inhibitors, etc.; (2) patients who did not have enhanced abdominal computed tomography (CT) or magnetic resonance imaging (MRI) images before baseline, or the third lumbar vertebra (L3) was not within the imaging range; (3) baseline blood routine, c-reactive protein(CRP), and alpha-fetoprotein(AFP) were not performed within two weeks before treatment; and (4) having other malignancies or combined severe extrahepatic disease.

The study was conducted by the Declaration of Helsinki, and experienced clinicians determined patient eligibility for combined therapy based on guidelines. In addition, this study was approved by the Ethics Committee of Beijing Ditan Hospital, Capital Medical University (JDLKZ 2021-056-01).

### Image analysis

**Figure 1:**
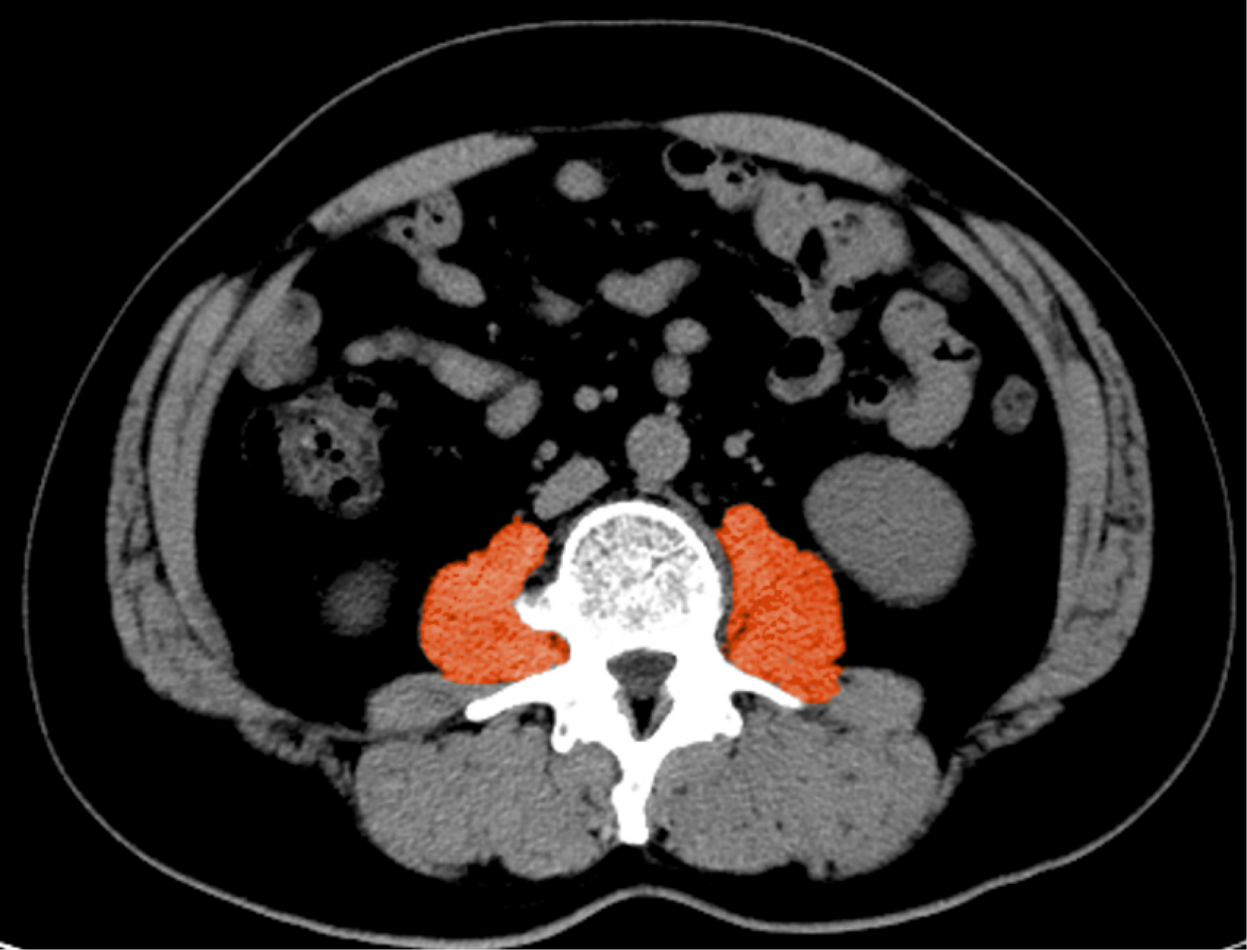
Measurement of the cross-sectional area of the psoas muscle at the L3 vertebral layer.

CT or MRI before the first dose of PD-1 inhibitors was used to measure PMI and was independently assessed by two radiologists, and disagreements were resolved by a third experienced radiologist. The cross-sectional area of the psoas muscle was measured at the level of the L3. Axial images at the level of L3 were manually measured on a dedicated workstation (SliceOmatic software, version 5.0) for specific tissues (− 29 to + 150 Hounsfield units (HU) thresholds) (**Figure 1**). PMI was determined by dividing L3 psoas cross-sectional area (mm^2^) by height squared (m^2^) (26). Given the absence of a standardized criterion for sarcopenia in China, we employed X-tile software (version 3.6.1) to determine the optimal cut-off value for psoas muscle index (PMI), which was separately selected based on gender to account for inherent gender disparities (20, 27). The patient cohort was divided into low and normal PMI cohorts, and patients in the low PMI cohort were considered to be in a state of sarcopenia. ΔPMI indicates the difference between baseline PMI and PMI at week four, showing dynamic changes in PMI after treatment.

### Treatment regimen

Lenvatinib, in combination with PD-1 inhibitors, was administered to patients with unresectable HCC who met the criteria, and the treatment regimens are summarized below. Lenvatinib was orally administered daily, with the initial dose determined according to body weight (≥ 60 kg, 12 mg; < 60 kg, 8 mg); PD-1 inhibitor was intravenously injected at 200 mg every three weeks, and the drugs used included sintilimab, camrelizumab, and tislelizumab. This combination regimen has been used as the first line of therapy for included patients.

Patients were followed every 2-3 months until death or the end of the study. OS and PFS were calculated from the initiation of the PD-1 inhibitor. At the same time, ORR and disease control rate (DCR) were obtained based on the best radiographic response observed during treatment. ORR was defined as the proportion of patients achieving complete response (CR) and partial response (PR) according to modified Response Evaluation Criteria in Solid Tumors (mRECIST). At the same time, DCR increased the proportion of patients with stable disease (SD).

### Variable collection

We retrospectively collected patients’ baseline data, including age, sex, etiology, previous treatment history, and BCLC stage. BCLC stage combines multiple information, including tumor size, number, ECOG PS, portal vein tumor thrombus (PVTT), extrahepatic metastases, and Child-Pugh class. In addition, the following parameters were recorded and calculated: neutrophil-to-lymphocyte ratio (NLR), platelet-to-lymphocyte ratio (PLR), albumin-bilirubin (ALBI), prognostic nutritional index (PNI), AFP, and CRP. Individual measures such as NLR, PLR, PNI, CRP, and PMI were used as continuous variables to draw the receiver operating curve (ROC) for predicting the 1-year survival of patients and to compare AUC. The cut-off values of the above continuous variables were determined using X-tile software and subsequently categorized into binary classification indicators.

### Statistical analysis

In this study, R software (version 4.1.3, http://www.rproject.org) was performed for data analysis, and P < 0.05 was judged to be statistically different. Demographic data and disease characteristics were compared between patients in the low and normal PMI groups, continuous data were expressed as mean plus or minus standard deviation or median (interquartile range), and T-test and Mann-Whitney U test were used to compare the two groups. Categorical data were described as numbers (percentages) and compared using the Chi-square test. PFS and OS were calculated using the Kaplan-Meier method, and differences between groups were tested using the Log-Rank test. Cox multivariate analysis was performed to investigate important predictive variables affecting prognosis, and nomogram-based prediction models were constructed. In addition, the ROC and calibration curve were plotted separately to demonstrate the model’s performance, and the predictive value between different variables was compared by calculating the area under the ROC curve (AUC).

## Results

### Patient characteristics

A total of 180 patients were enrolled in this study, and **Table 1** documents the patient baseline characteristics of the overall cohort. Most patients were male (n = 151, 83.9%), with a median age of 57.5 [51.0, 64.0] years. Hepatitis B virus (HBV) infection was the leading cause in this cohort (n = 159, 88.3%). Most patients had Child-Pugh A liver function before initiating PD-1 inhibitor (n = 127, 70.6%). Approximately half of the patients have an ECOG PS score of 1/2 (n = 94, 52.2%), along with PVTT (n = 100, 55.6%) and distant metastasis (n = 100, 55.6%). Moreover, 144 (80.0%) patients had BCLC stage C. Previous surgical resection, TACE, and ablation were performed in 29 (16.1%), 158 (87.8%), and 74 (41.1%) patients, respectively.

**Table 1:** Baseline characteristics.

Characteristics	Overall (n=180)	low PMI (n=46)	normal PMI (n=134)	P
Age (median [IQR])	57.5 [51.0, 64.0]	60.0 [55.0, 65.0]	56.5 [48.0, 64.0]	0.0138
Gender, n(%)				0.6719
Male	151 (83.9)	40 (87.0)	111 (82.8)	
Female	29 (16.1)	6 (13.0)	23 (17.2)	
ECOG, n(%)				0.0589
PS 0	86 (47.8)	28 (60.9)	58 (43.3)	
PS 1/2	94 (52.2)	18 (39.1)	76 (56.7)	
Etiology, n(%)				0.5191
HBV	159 (88.3)	41 (89.1)	118 (88.1)	
HCV	13 (7.2)	2 (4.3)	11 (8.2)	
Others	8 (4.4)	3 (6.5)	5 (3.7)	
Prior regimens, n(%)				
Surgery	29 (16.1)	5 (10.9)	24 (17.9)	0.3744
TACE	158 (87.8)	38 (82.6)	120 (89.6)	0.3272
Ablation	74 (41.1)	12 (26.1)	62 (46.3)	0.026
AFP (%)				0.2711
<400	112 (62.2)	25 (54.3)	87 (64.9)	
≥400	68 (37.8)	21 (45.7)	47 (35.1)	
Number, n(%)				0.2026
<3	71 (39.4)	14 (30.4)	57 (42.5)	
≥3	109 (60.6)	32 (69.6)	77 (57.5)	
Size, n(%)				0.9568
<5cm	107 (59.4)	28 (60.9)	79 (59.0)	
≥5cm	73 (40.6)	18 (39.1)	55 (41.0)	
PVTT, n(%)	100 (55.6)	29 (63.0)	71 (53.0)	0.3113
Metastasis, n(%)	75 (41.7)	23 (50.0)	52 (38.8)	0.2479
Child-Pugh, n(%)				0.0632
Class A	127 (70.6)	27 (58.7)	100 (74.6)	
Class B	53 (29.4)	19 (41.3)	34 (25.4)	
BCLC, n(%)				0.7649
Stage B	36 (20.0)	8 (17.4)	28 (20.9)	
Stage C	144 (80.0)	38 (82.6)	106 (79.1)	
NLR (median [IQR])	2.9 [2.0, 4.1]	3.2 [2.0, 4.1]	2.8 [2.0, 4.1]	0.7
PLR (median [IQR])	116.3 [78.3, 166.0]	133.8 [76.6, 198.3]	114.3 [79.2, 154.4]	0.1963
PNI (median [IQR])	42.5 [37.4, 47.0]	41.5 [36.5, 44.4]	43.1 [39.3, 47.2]	0.0215
CRP (median [IQR])	8.4 [2.2, 23.2]	21.0 [7.4, 45.6]	6.3 [1.9, 16.6]	0.0001

### Assessment of sarcopenia

**Figure 2:**
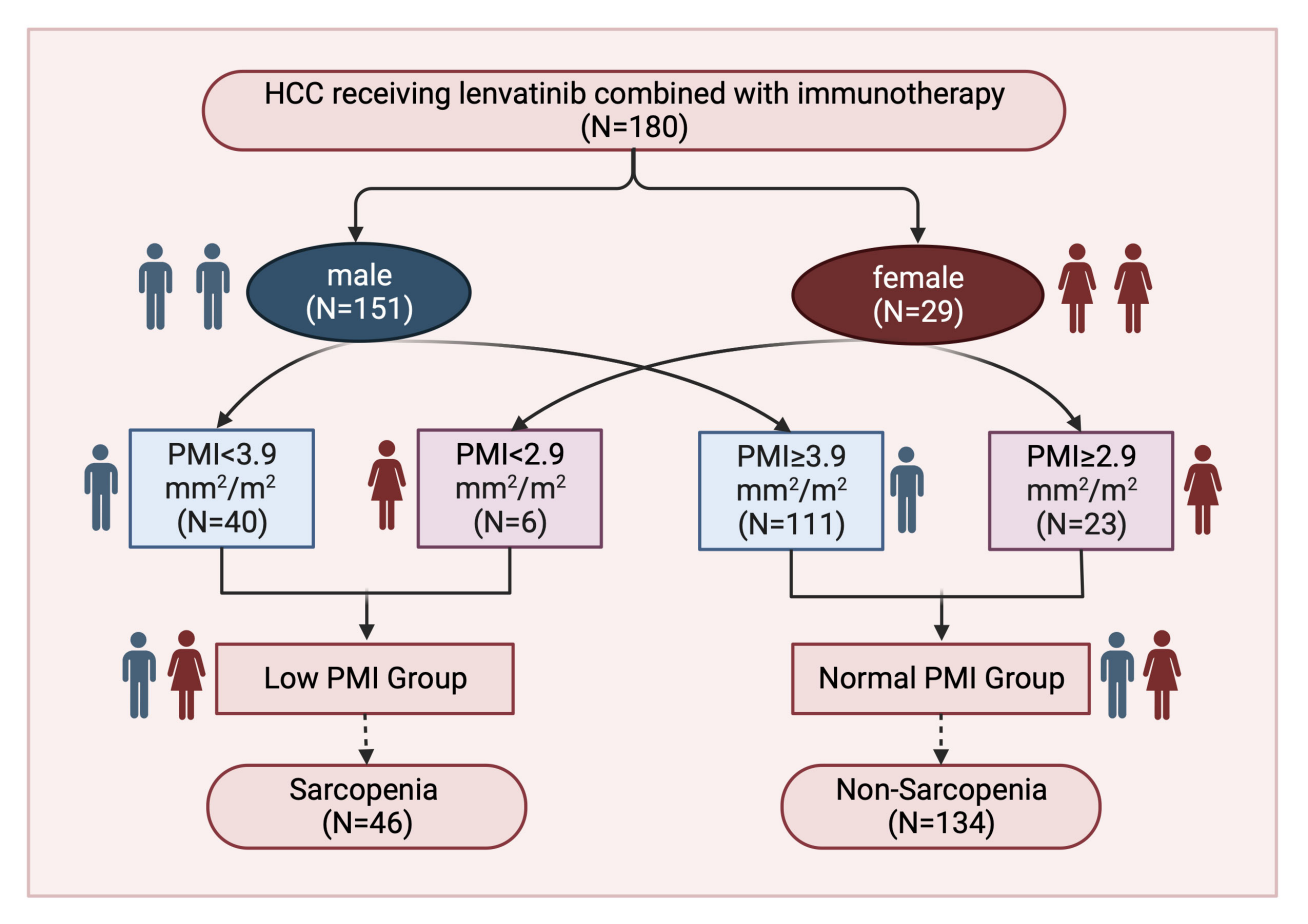
Study flowchart of the current study.

The patient cohort consisted of 151 males and 29 females, for whom separate cut-off values were calculated based on gender. A PMI cut-off of 2.9 mm^2^/m^2^ was determined for females, assigning 6 patients to the low PMI cohort and 23 patients to the normal PMI cohort. In contrast, males had a PMI cut-off of 3.9 mm^2^/m^2^, classifying 40 patients into the low PMI group and 111 patients into the normal PMI group. Ultimately, 46 (25.6%) patients were considered to be in a state of sarcopenia (**Figure 2**). **Table 1** also presents patient characteristics for the normal and low PMI groups, facilitating a comparison of baseline differences between these two cohorts. Patients with sarcopenia were older (median 60.0 vs. 56.5, p = 0.0138), exhibited elevated serum CRP levels (median 21.0 vs. 6.3, p = 0.0001), demonstrated a lower frequency of prior ablations (26.1% vs. 46.3%, p = 0.026), and displayed reduced PNI levels (median 41.5 vs 43.1, p = 0.0215) compared to non-sarcopenic patients.

### Sarcopenia and treatment response and survival

Among the overall population, 62 (34.4%) patients developed decreased PMI levels after four weeks of PD-1 inhibitor therapy, but interestingly two patients exhibited an elevation in PMI. Evaluation based on mRECIST criteria revealed CR in 9 patients (5.0%), PR in 38 patients (21.1%), SD in 83 patients (46.1%), and progressive disease (PD) in 50 patients (27.8%). Both of the two patients with increased PMI got an objective response as indicated by PR. The ORR and DCR were 26.1% and 72.2%, respectively. The patients with low PMI had significantly lower rates of ORR and DCR compared to those with normal PM, 4.3% vs 33.6% (p = 0.0002) and 39.1% vs 83.6% (p < 0.0001), respectively.

**Figure 3:**
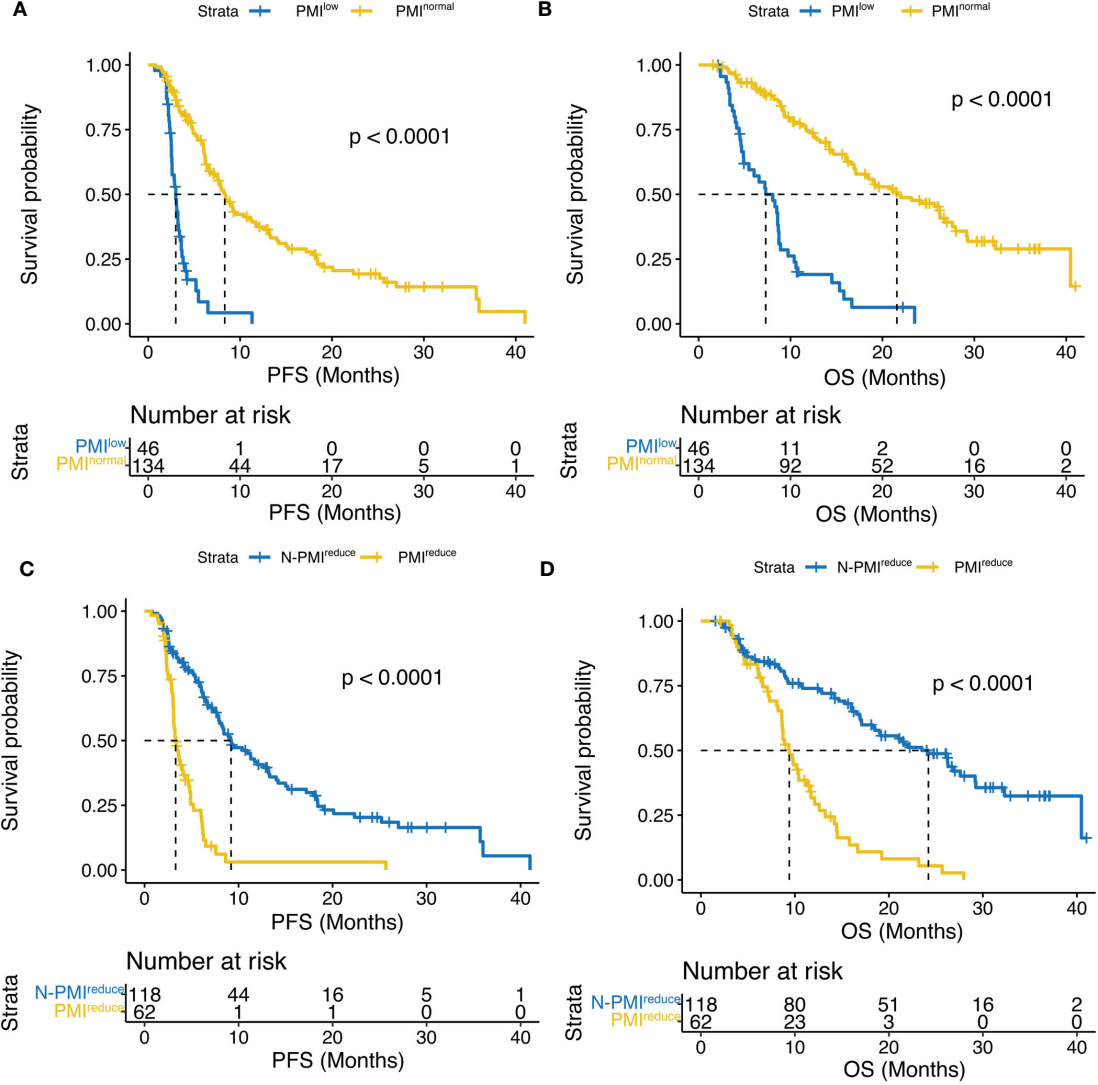
Kaplan–Meier curves compared **(A)** PFS and **(B)** OS according to PMI, and **(C)** PFS and **(D) **OS according to ΔPMI. PFS, progression-free survival; OS,overall survival; PMI, psoas muscle index; ΔPMI, PMI reduce after four weeks of treatment.

As of January 2023, 111 (61.7%) patients had died, and 135 (75.0%) had a progression event. Median PFS and median OS were 6.2 (95% CI, 5.5 – 7.9) months and 16 (95% CI, 13.2 – 19.2 months, respectively. According to sex-specific cut-offs, patients with sarcopenia had a significantly worse PFS compared to those without sarcopenia, 3.0 months [95% CI, 2.57 – 3.6] vs 8.3 months [95% CI, 6.7 – 11.3], respectively (HR, 0.18; 95% CI, 0.12-0.28; p < 0.0001). A similar difference was found in the median OS, 7.3 months (95% CI, 4.9 – 8.7) for the sarcopenia group and 21.6 months (95% CI, 17.1 – 27.6) for the non-sarcopenia group, HR, 0.19; 95% CI, 0.13-0.3; p < 0.0001. (**Figures 3A, B**).

In addition, we analyzed the relationship between PMI change and survival after four weeks of treatment. Notably, patients with no significant change in PMI exhibited a significantly longer median PFS compared to those with decreased PMI (9.2 months vs. 3.3 months, p < 0.0001) (**Figure 3C**). Furthermore, this difference was observed in OS (24.2 months vs 9.4 months, p < 0.0001) (**Figure 3D**).

### Serum biomarkers and survival

**Figure 4:**
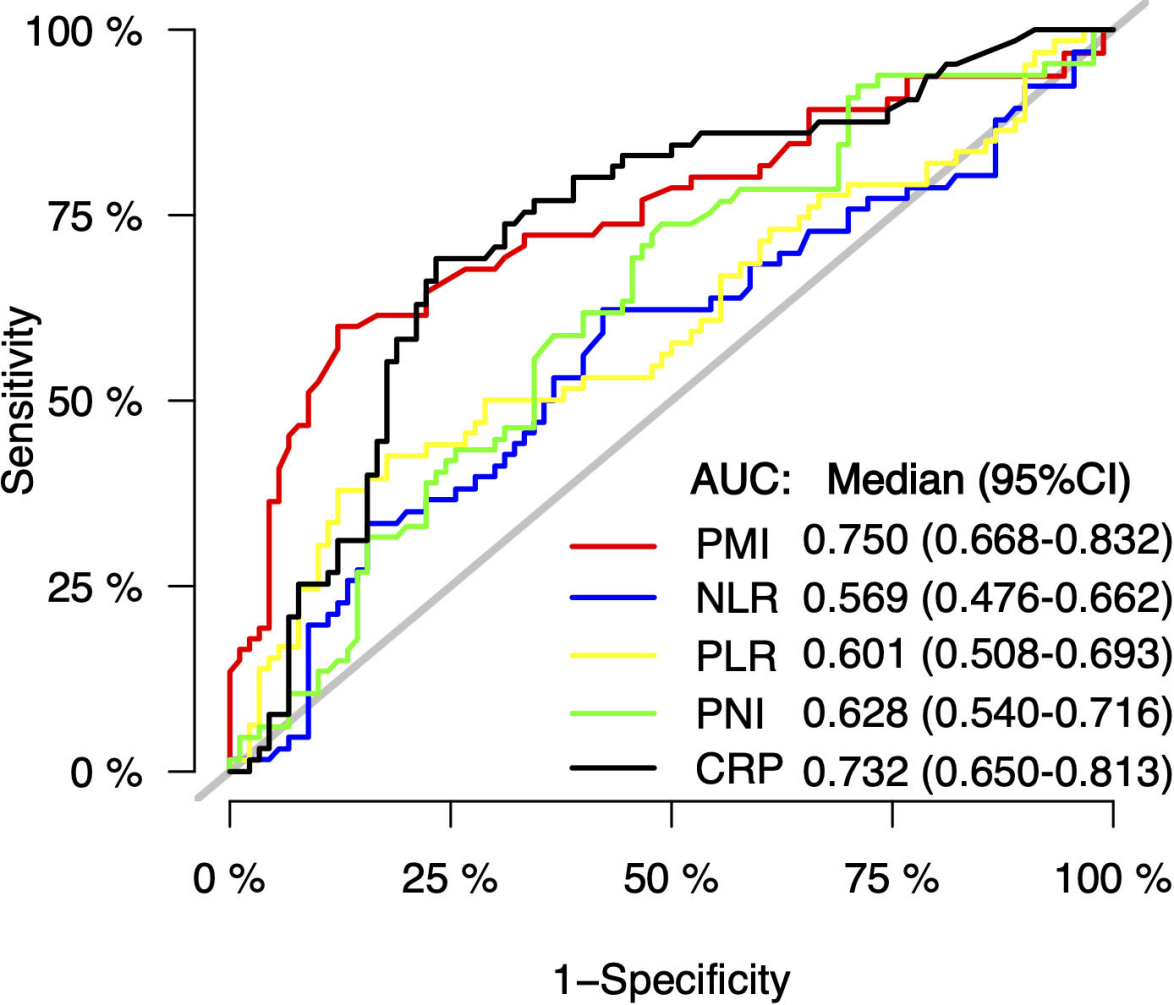
The discriminatory power of PMI, NLR, PLR, PNI or CRP on the receiver operating characteristic (ROC) curves to predict the survival. AUC, areas under the ROC curve; NLR, neutrophil-to-lymphocyte ratio; PLR, platelet-to-lymphocyte ratio; PNI, prognostic nutritional index; CRP, C-reactive protein.

**Figure 5:**
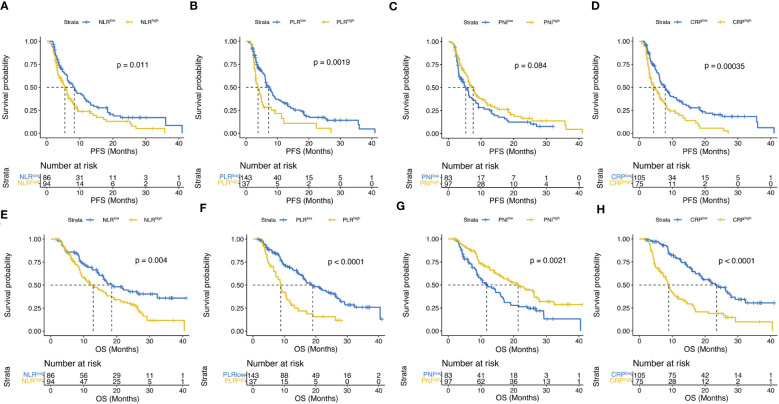
Kaplan–Meier curves compared PFS **(A–D)** according to NLR, PLR, PNI or CRP and OS **(E–H) **according to NLR, PLR, PNI or CRP.

Multiple inflammatory nutritional indicators currently exist to assess patient prognosis, and the ROC curves in **Figure 4** describe the discriminatory power of PMI, NLR, PLR, PNI, and CRP for patient survival prediction, with AUC values of 0.750 (95% CI, 66.8 – 83.2), 0.569 (95% CI, 0.476 – 0.662), 0.601 (95% CI, 0.508 – 0.693), 0.628 (95% CI, 0.540 – 0.716), and 0.732 (95% CI, 0.650 – 0.813), respectively. The cut-off values for continuous variables were determined by X-tile software, and the ideal cut-off values for NLR, PLR, PNI, and CRP were 2.8, 180.3, 42.1, and 9.9 mg/L, respectively. The above cut-off divided the patient cohort into two groups, and survival differences were almost observed in both PFS and OS (**Figure 5**).

### Predictive model

**Tables 2**, **3** indicate the results of Cox regression for OS and PFS, respectively. Univariate analysis of OS revealed significant associations between patient death and various factors, including Child-Pugh class, BCLC stage, previous surgical resection, history of TACE and ablation, tumor number, PVTT, distant metastasis, NLR, PLR, PNI, AFP, CRP, and PMI. Subsequent multivariate analysis identified independent prognostic factors for OS as the presence of distant metastasis (HR, 1.63; 95% CI, 1.01 – 2.62; p = 0.0445), high PLR (HR, 1.61; 95% CI, 1.01 – 2.56; p = 0.0459), high CRP (HR, 1.73; 95% CI, 1.07 – 2.79; p = 0.0251), and normal PMI (HR, 0.26; 95% CI, 0.16 – 0.41; p < 0.0001). Next, we conducted a PFS-related analysis for HCC treated with the combination of lenvatinib and PD-1 inhibitor therapy, and univariate results showed tumor number, NLR, PLR, PNI, CRP, and PMI as potential predictors of PFS. However, only sarcopenia was considered a significant independent risk factor for progression in multivariate analysis (HR, 0.22; 95% CI, 0.14 – 0.35; p < 0.0001).

**Table 2:** Cox regression for OS.

Characteristic	Univariate analysis		Multivariate analysis	
	HR (95%CI)	P	HR (95%CI)	P
PMI (normal vs low)	0.19 (0.13-0.3)	<0.001	0.26 (0.16-0.41)	<0.001
Gender (male vs female)	1.31 (0.75-2.3)	0.345		
Age (≥63 vs <63)	0.73 (0.48-1.11)	0.143		
ECOG (PS1/2 vs PS0)	0.75 (0.51-1.09)	0.127		
Surgery (yes vs no)	0.62 (0.37-1.06)	0.083	0.98 (0.56-1.73)	0.9508
TACE (yes vs no)	0.35 (0.21-0.57)	<0.001	0.57 (0.32-1.03)	0.0627
Ablation (yes vs no)	0.53 (0.36-0.79)	0.002	0.94 (0.59-1.49)	0.7788
Number (≥3 vs <3)	1.71 (1.15-2.55)	0.008	1.49 (0.96-2.32)	0.0753
Size (≥5cm vs <5cm)	0.8 (0.55-1.18)	0.258		
PVTT (yes vs no)	1.48 (1.01-2.16)	0.044	1.59 (0.96-2.63)	0.0736
Metastasis (yes vs no)	1.94 (1.33-2.83)	0.001	1.63 (1.01-2.62)	0.0445
Child-Pugh Class (B vs A)	1.54 (1.04-2.29)	0.032	0.73 (0.45-1.19)	0.2014
BCLC Stage (C vs B)	1.79 (1.04-3.1)	0.036	0.75 (0.35-1.61)	0.4606
AFP (≥400 vs <400)	1.59 (1.09-2.32)	0.017	1.38 (0.92-2.07)	0.1193
NLR (high vs low)	1.74 (1.19-2.55)	0.005	0.97 (0.61-1.53)	0.8837
PLR (high vs low)	2.45 (1.6-3.74)	<0.001	1.61 (1.01-2.56)	0.0459
PNI (high vs low)	0.56 (0.38-0.81)	0.002	0.67 (0.43-1.06)	0.0847
CRP (high vs low)	2.87 (1.97-4.19)	<0.001	1.73 (1.07-2.79)	0.0251

**Table 3:** Cox regression for PFS.

Characteristic	Univariate analysis		Multivariate analysis	
	HR (95%CI)	P	HR (95%CI)	P
PMI (normal vs low)	0.18 (0.12-0.28)	<0.001	0.22 (0.14-0.35)	<0.001
Gender (male vs female)	1.01 (0.66-1.56)	0.95		
Age (≥63 vs <63)	0.87 (0.6-1.26)	0.458		
ECOG (PS1/2 vs PS0)	0.91 (0.65-1.29)	0.608		
Surgery (yes vs no)	0.68 (0.42-1.1)	0.117		
TACE (yes vs no)	0.83 (0.48-1.42)	0.494		
Ablation (yes vs no)	0.87 (0.62-1.23)	0.426		
Number (≥3 vs <3)	1.58 (1.11-2.24)	0.012	1.39 (0.97-2)	0.0766
Size (≥5cm vs <5cm)	1.09 (0.77-1.53)	0.637		
PVTT (yes vs no)	1.31 (0.93-1.84)	0.127		
Metastasis (yes vs no)	1.16 (0.82-1.63)	0.411		
Child-Pugh Class (B vs A)	1.31 (0.9-1.91)	0.152		
BCLC Stage (C vs B)	1.01 (0.66-1.56)	0.95		
AFP (≥400 vs <400)	1.26 (0.88-1.8)	0.199		
NLR (high vs low)	1.56 (1.1-2.2)	0.012	1.2 (0.81-1.78)	0.3704
PLR (high vs low)	1.87 (1.25-2.8)	0.002	1.25 (0.8-1.96)	0.3219
PNI (high vs low)	0.74 (0.53-1.04)	0.085	1.03 (0.71-1.49)	0.8878
CRP (high vs low)	1.88 (1.32-2.66)	<0.001	1.38 (0.92-2.07)	0.118

**Figure 6:**
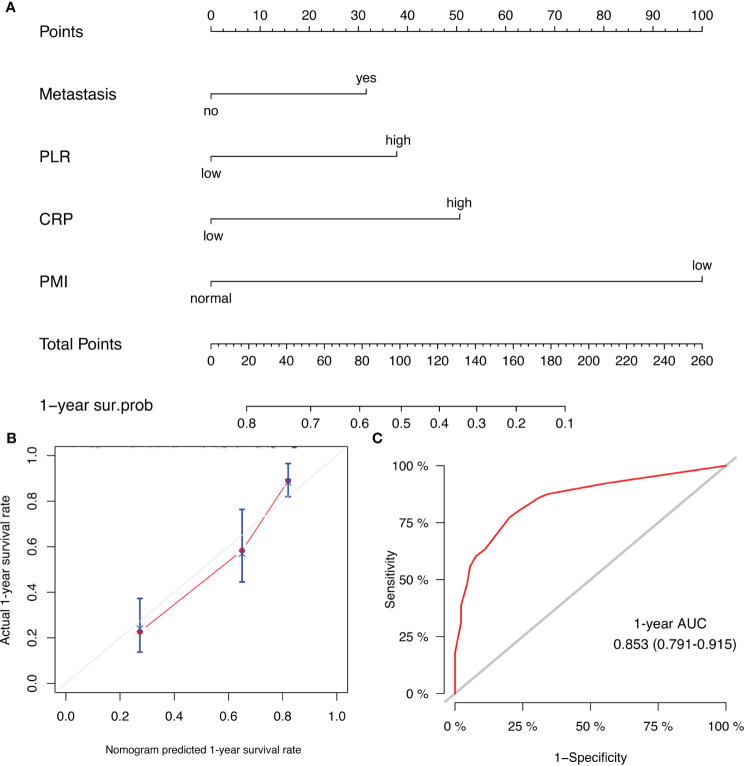
**(A)** Prognostic nomogram for HCC patients to assign their probability of survival at 1-year after lenvatinib plus PD-1 inhibitor treatment initiation; **(B)** Calibration curves of the nomogram at 1-year survival rate. **(C)** ROC curve of the nomogram at 1-year survival rate.

Prognostic models of patient survival were constructed using the four variables selected by Cox regression above. Nomograms were plotted to predict the probability of patient survival at one year (**Figure 6A**). Calibration curves showed good agreement between the model-predicted 1-year survival of patients and actual observations (**Figure 6B**). The discrimination of this model in the ROC curve was fair, with an AUC value of 0.853 (95% CI, 0.791 – 0.915) (**Figure 6C**).

### Adverse events

Treatment-emergent AEs are detailed in **Table 4**. In this study, the majority of patients (83.33%, n=180) experienced AEs, with 50% (n=90) reporting treatment-emergent AEs ≥ grade 3. Grade 3 or higher AEs were observed in 34 (73.91%) patients in the sarcopenia group and 56(41.79%) patients in the non-sarcopenia groups, indicating a significantly greater severity of AEs in the sarcopenia group compared to the non-sarcopenia group. In the sarcopenia group, four patients experienced immune-related adverse events (irAEs), including immune-related adrenal insufficiency, grade 3-4 ALT/AST elevation, and immune-related rash. Among the common treatment-emergent adverse events, fatigue (51.17% vs. 29.85%), elevated blood bilirubin (43.48% vs. 26.87%), and hypothyroidism (36.6% vs. 13.43%) were significantly different between groups. In addition, noteworthy disparities were noted in reported dose reductions (23.9% vs. 10.4%) or discontinuations (21.7% vs 5.2%) for the combined administration of lenvatinib and PD-1 inhibitor.

**Table 4:** Adverse events.

	Non-Sarcopenia Group (n=134)	Sarcopenia Group (n=46)	P-value
Total treatment-emergent AEs Total treatment-related treatment-emergent AEs	108 (80.59%) 108 (80.59%)	42 (91.3%) 42 (91.3%)	0.093 0.093
Treatment-emergent AEs (grade ≥3) Treatment-related treatment-emergent AEs (grade ≥3)	56 (41.79%) 52 (38.81%)	34(73.91%) 33 (71.74%)	<0.001 <0.001
Serious treatment-emergent AEs Serious treatment-related treatment-emergent AEs SAEs (grade 5)	4(2.99%) 3(2.24%) 0	4(8.70%) 4(8.70%) 0	0.105 0.051
IrAE Grade 3-4	3*	4**	0.051
The common treatment-emergent adverse events of either group			
Nausea/ Vomiting Any grade Grade ≥3	47 (35.07%) 4 (2.99%)	21 (45.65%) 8 (17.39%)	0.202 0.001
Fatigue Any grade Grade ≥3	40 (29.85%) 4 (2.99%)	24 (51.17%) 1 (2.17%)	0.006 1
Increased blood bilirubin Any grade Grade ≥3	36 (26.87%) 1(0.75%)	20 (43.48%) 1(2.17%)	0.036 0.425
Decreased serum albumin Any grade Grade ≥3	37(27.61%) 7 (5.22%)	19 (41.3%) 4(8.70%)	0.083 0.396
Hypertension Any grade Grade ≥3	33(24.63%) 2 (1.49%)	14 (30.43%) 0	0.439 1
Diarrhea Any grade Grade ≥3	22 (16.42%) 0	13(28.26%) 0	0.08
Ascites Any grade Grade ≥3	27 (20.15%) 2 (1.49%)	13 (28.26%) 3 (6.51%)	0.254 0.106
Hypothyroidism Any grade Grade ≥3	18 (13.43%) 0	17 (36.6%) 0	0.001
Dose reduction Nausea/Vomiting Fatigue Hepatic encephalopathy Rash Palmar-plantar erythrodysesthesia Grade 3 hypertension	14 (10.4%) 4 4 2 2 0 2	11 (23.9%) 8 1 0 1 1 0	0.023
Discontinuation Proteinuria Fever Nausea/ Vomiting Pancreatitis Pneumonia Adrenal Insufficiency Upper Gastrointestinal Hemorrhage Grade 3 increased blood bilirubin	7 (5.2%) 0 0 3 2 1 0 0 1	10 (21.7%) 1 2 4 0 0 1 1 1	0.001

ALT, alanine aminotransferase; AST, aspartate aminotransferase; AE, adverse events.

*1 case had grade 3-4 ALT/AST elevation,1 case reported immune-related rash, and 1 case reported interstitial pneumonia.

**2 case reported immune-related adrenal insufficiency, 1 case had grade 3-4 ALT/AST elevation, 1 case reported immune-related rash.

## Discussion

In China, the combination of lenvatinib and PD-1 inhibitors is the most commonly first-line treatment regimen in advanced HCC patients. However, the response to this combination regimen varies, underscoring the need for reliable biomarkers to predict treatment outcomes. Our study integrates PMI, inflammation, and nutritional indicators to explore the relationship between sarcopenia and clinical outcomes in HCC patients treated with the combination of lenvatinib and PD-1 inhibitors. Our analysis reveals that lower PMI is consistently linked to poorer prognoses, both at baseline and for those developing sarcopenia during treatment. Previous studies in this field have shown some controversy. Our findings also differed from the research on advanced HCC patients treated with ATEZ/BEV, where sarcopenia does not determine PFS or OS (25). However, some studies demonstrated that initial skeletal muscle status affects the prognosis of TKIs (sorafenib or lenvatinib) (16, 17) or ATEZ/BEV (23). This discrepancy may stem from smaller sample sizes and a different indicator of sarcopenia (SMI or PMI). Besides, Similar observations have been made in meta-analyses and follow-up studies (10, 14–17, 21, 28), suggesting the clinical significance of sarcopenia in advanced HCC.

The precise mechanisms behind the adverse effects of low PMI on HCC treatment and prognosis are not fully understood but seem to be linked to the tumor microenvironment (inflammation and immunity) and cytokine activity. Skeletal muscle, acting as an immune modulator, mitigates the harmful impact of pro-inflammatory adipokines by producing myokines like Interleukin-15, contributing to the tumor microenvironment (29–32). Interleukin-15 elevates the population of circulating NK cells and CD8^+^ T cells, potentially augmenting the efficacy of ICIs (33–35). Muscle atrophy, accompanied by reduced secretion of myokines, may affect immune cell functionality and quantity, thereby fostering systemic inflammation and immune dysregulation. Studies indicate a substantial decrease in peripheral blood CD3+ and CD4+ T cell counts in HCC patients with sarcopenia (36). Systemic inflammation is pivotal in promoting malignant cell proliferation, invasion, and metastasis (37–40). Increased inflammatory markers in individuals with sarcopenia support the idea that sarcopenia reflects heightened metabolic activity, leading to systemic inflammation and muscle depletion (41).

Our study used four variables associated with OS (metastasis, PLR, CRP, and PMI) to construct nomograms. AFP is not an independent predictive factor of OS, so it was not included in the construction of the prediction model. The previous model we developed for predicting the efficacy of combined TKIs and ICI regimens in unresectable HCC did not account for the impact of CRP levels and skeletal muscle loss (41). This current study serves as a valuable addition to our prior research. The prognostic nomograms exhibited ample discriminative ability within the cohort and effectively predicted overall survival in HCC patients undergoing combined lenvatinib and PD-1 inhibitor therapy (C-index: 0.853). The calibration curve for 12-month post-treatment overall survival probability demonstrated optimal concurrence between predicted and observed outcomes. Furthermore, the ROC curve illustrated the discriminative power (AUC: 0.750) of PMI, NLR, PLR, PNI, and CRP. The results suggest a significant association between inflammatory biomarkers and tumor response in HCCs. Skeletal muscle loss, serum AFP, and CRP levels have been identified as potential predictors of overall survival and tumor response in unresectable HCC patients undergoing the combination of TKIs and PD-1 inhibitors (42). However, their study did not consider the impact of other inflammatory and nutritional indicators. Incorporating these enhancements is anticipated to improve the performance of our predictive model, potentially facilitating its applications in clinical settings following further validation.

Our study demonstrates a significant association between low PMI during the treatment of lenvatinib and PD-1 inhibitor and PFS. Patients with low PMI had higher age and CRP levels compared to those with normal PMI, and consistent with prior research (12, 43). Tada et al. (44) found that no significant differences in OS and PFS between older and younger HCC patients treated with ATEZ/BEV. Based on the exclusion of active infection at the time of admission, elevated CRP levels may be associated with the tumor. Furthermore, our multivariable analysis results revealed no significant associations between age or CRP and PFS. These findings suggests that the imbalance of baseline age and CRP in the two groups did not impact the prognosis assessment.

In our cohort, HCC patients with sarcopenia had a significantly worse prognosis and lower anti-tumor efficacy, with ORR of 4.3% and mOS of 7.3 months, which were lower than 12.6 months reported in the sarcopenia cohort treated by ATEZ/BEV (42). Despite only 29.4% of HCC patients having Child-Pugh B classification, their prognosis was still poorer compared to the mOS of 13.8 months observed in mono-lenvatinib treatment (42). The incidence of grade ≥3 AEs was higher in sarcopenia patients(73.91% vs. 41.79%), potentially attributed to their diminished drug tolerance towards agents such as lenvatinib and PD-1 inhibitors. Additionally, dysfunction in direct pathways, such as alterations in the phosphatidylinositol-3-kinase/AKT-mammalian target of rapamycin (PI3K/AKT- mTOR) pathway, which plays a pivotal role in muscle protein synthesis, had been observed (14). The pathway of PI3K/AKT- mTOR will also lead to tumor progression (45). The high rate of dose reductions (23.9% vs. 10.4%) or discontinuations (21.7% vs 5.2%) may contribute to the unfavorable outcome in sarcopenia patients. Thus, HCC patients with sarcopenia are more susceptible to the impact of treatment-related AEs, which not only challenges treatment efficacy but also increases the complexity of the therapeutic approach. Notably, the advanced age of sarcopenic HCC patients in our study may contribute to the increased incidence of AEs. Furthermore, it is noteworthy that two patients exhibited an elevation in PMI during the course of treatment, which was concomitant with objective response as indicated by PR. Despite being diagnosed with HCC, one of these two patients persisted in engaging in resistance training as part of his habitual routine. These findings imply that this therapeutic regimen may benefit less for individuals with HCC and sarcopenia compared to non-sarcopenic patients. Furthermore, in our study the patients with a decrease in PMI following 4 weeks of treatment had adverse response, supporting the notion of treatment-induced skeletal muscle malnutrition or PMI reduction leading to poorer prognosis and immunotherapy resistance. Thereby, the potential for enhancing treatment response through optimization of skeletal muscle nutritional status is encouraged.

Our study had certain limitations. Firstly, the sample size was limited, and all data were retrospectively analyzed from a single center, potentially introducing selection bias. Secondly, we could not assess other potential factors related to sarcopenia, such as the amount of physical activity, dietary habits, or the presence of other metabolic diseases. Future investigations should validate our findings in a more extensive population and consider the potential impacts of nutritional and exercise intervention strategies. Moreover, incorporating a more comprehensive range of pertinent variables can enhance predictive models and augment their clinical applicability.

## Conclusion

Sarcopenia was significantly associated with poor clinical outcomes, including PFS and OS, in HCC patients treated combined with lenvatinib and PD-1 inhibitor therapy. The predictive nomogram combining inflammatory markers and nutritional status is expected to be used in clinical practice after further validation.

## Data availability statement

The raw data supporting the conclusions of this article will be made available by the authors, without undue reservation.

## Ethics statement

The studies involving humans were approved by Capital Medical University Affiliated Beijing Ditan Hospital Ethics Committee. The studies were conducted in accordance with the local legislation and institutional requirements. The participants provided their written informed consent to participate in this study. Written informed consent was obtained from the individual(s) for the publication of any potentially identifiable images or data included in this article.

## Author contributions

WS: Data curation, Writing – original draft, Writing – review & editing. XY: Data curation, Writing – original draft, Writing – review & editing. XL: Visualization, Writing – original draft. JW: Visualization, Writing – original draft. MY: Visualization, Writing – original draft. WL: Writing – original draft, Writing – review & editing. XD: Conceptualization, Writing – review & editing. JC: Conceptualization, Writing – review & editing.
